# Novel Gammaherpesvirus Infections in Narrow-Ridged Finless Porpoise (*Neophocaena asiaeorientalis*) and False Killer Whales (*Pseudorca crassidens*) in the Republic of Korea

**DOI:** 10.3390/v16081234

**Published:** 2024-07-31

**Authors:** Sung Bin Lee, Kyung Lee Lee, Sang Wha Kim, Won Joon Jung, Da Sol Park, Seyoung Lee, Sib Sankar Giri, Sang Guen Kim, Su Jin Jo, Jae Hong Park, Mae Hyun Hwang, Eun Jae Park, Jong-pil Seo, Byung Yeop Kim, Se Chang Park

**Affiliations:** 1 Laboratory of Aquatic Biomedicine, College of Veterinary Medicine and Research Institute for Veterinary Science, Seoul National University, Seoul 08826, Republic of Korea; lsbin1129@snu.ac.kr (S.B.L.); cwj0125@snu.ac.kr (W.J.J.); dabol2@snu.ac.kr (D.S.P.); ssgiri@snu.ac.kr (S.S.G.); ssjjone@snu.ac.kr (S.J.J.); jaehong139@snu.ac.kr (J.H.P.); ghkdao@snu.ac.kr (M.H.H.); eunjae.p@snu.ac.kr (E.J.P.); 2 Cetacean Research Institute, National Institute of Fisheries Science, Ulsan 44780, Republic of Korea; moby19@korea.kr; 3 College of Veterinary Medicine & Institute of Veterinary Science, Kangwon National University, Chuncheon 24341, Republic of Korea; sangwhakim@kangwon.ac.kr; 4 College of Veterinary Medicine and Veterinary Medical Research Institute, Jeju National University, Jeju 63243, Republic of Korea; yaya4954@daum.net (S.L.); jpseo@jejunu.ac.kr (J.-p.S.); 5 Department of Biological Sciences, Kyonggi University, Suwon 16227, Republic of Korea; imagine0518@kyonggi.ac.kr; 6 Department of Marine Industry and Maritime Police, College of Ocean Science, Jeju National University, Jeju 63243, Republic of Korea

**Keywords:** herpesvirus, cetacean gammaherpesvirus, narrow-ridged finless porpoise, false killer whale

## Abstract

A female narrow-ridged finless porpoise (*Neophocaena asiaeorientalis*) stranded on a beach on Jeju Island showed epithelial proliferative skin lesions on its body. Two false killer whales (*Pseudorca crassidens*), caught using nets near Gangneung and Samcheok, respectively, had multiple plaques on their penile epidermis. Histological examination of the epidermis revealed that all the lesions had common features, including accentuated rete pegs, ballooning changes, and eosinophilic intranuclear inclusion (INI) bodies. Based on the histopathological results, herpesvirus infection was suspected, and thus further analysis was conducted using herpesvirus-specific primers. Based on nested polymerase chain reaction (PCR) tests using the herpesvirus-detectable primers, the PCR products demonstrated two fragments: a 222-base-pair (bp) sequence of the DNA polymerase gene, SNUABM_CeHV01, showing 96.4% identity with a bottlenose dolphin herpesvirus from the Jeju narrow-ridged finless porpoise; and a 222 bp sequence of the DNA polymerase gene, SNUABM_CeHV02, showing 95.95% identity with the same bottlenose dolphin herpesvirus from the Gangneung and Samcheok false killer whales. The significance of this study lies in its ability to demonstrate the existence of novel cetacean herpesviruses in South Korean seawater, representing an important step forward in studying potentially harmful pathogens that affect endangered whale and dolphin populations.

## 1. Introduction

Herpesviruses are widespread, characterized by large double-stranded DNA, and belong to the Herpesviridae family [1,2,3,4]. More than 130 herpesviruses have been identified, some of which have been reported in humans and other animals [5,6,7,8,9]. The Herpesviridae family is divided into three subfamilies, Alphaherpesvirinae, Betaherpesvirinae, and Gammaherpesvirinae, based on genomic structure and biological characteristics [10,11,12,13]. Herpesvirus infection is common in most vertebrates, including humans; however, cases of herpesvirus infection in cetaceans have rarely been reported [14,15,16]. In the case of cetacean herpesviruses, alpha- and gammaherpesviruses have been discovered, and a few of them cause dermatitis, such as Delphinid herpesvirus 3 (DeHV–3), 7, and 8 in bottlenose dolphins (*Tursiops truncatus*) [17,18] and *Stenella coeruleoalba* herpesvirus in striped dolphins (*S. coeruleoalba*) [19]. Cetacean alphaherpesviruses are mostly associated with skin lesions, whereas cetacean gammaherpesviruses are associated with genital and oral infections [17,20,21].

The narrow-ridged finless porpoise (*Neophocaena asiaeorientalis*) is a dominant coastal odontocete in South Korean waters [22,23,24]. The false killer whale (*Pseudorca crassidens*) is primarily distributed in the East Sea of South Korea, although the exact number of their population is not yet known [24,25]. Cetaceans are indicator species of ocean pollution and play a role in supplying phosphorus, one of the essential nutrients in the marine ecosystem, from the ocean floor to the surface [26,27,28]. However, more than 1000 narrow-ridged finless porpoises are caught by stow nets annually in South Korean waters, decreasing their population from 36,000 in 2005 [29] to 13,000 in 2011 [30]. Despite the risk of extinction, pathological research on narrow-ridged finless porpoises and false killer whales has rarely been conducted in the Republic of Korea. Further veterinary research is required for disease prevention and population control of narrow-ridged finless porpoises and false killer whales.

This study is the first to report cetacean gammaherpesvirus infections in South Korean seawater from a narrow-ridged finless porpoise and two false killer whales, providing the necropsy findings, histopathological features, and genomic analysis. Although novel alphaherpesvirus has been discovered in the lung tissue of a stranded false killer whale in Japan [31], research on cetacean herpesviruses is still limited in Asia. Specifically, herpesviruses infecting certain coastal dolphin or porpoise species have been widely reported in South America, Europe, and the United States, but are rarely reported in Asia. Diseases that particularly break out around the genital area, such as herpesviruses, can significantly impact the breeding of endangered cetacean species and therefore need to be treated with importance from a conservation medicine perspective.

## 2. Material and Methods

### 2.1. Necropsy and Sample Collection

**Figure 1:**
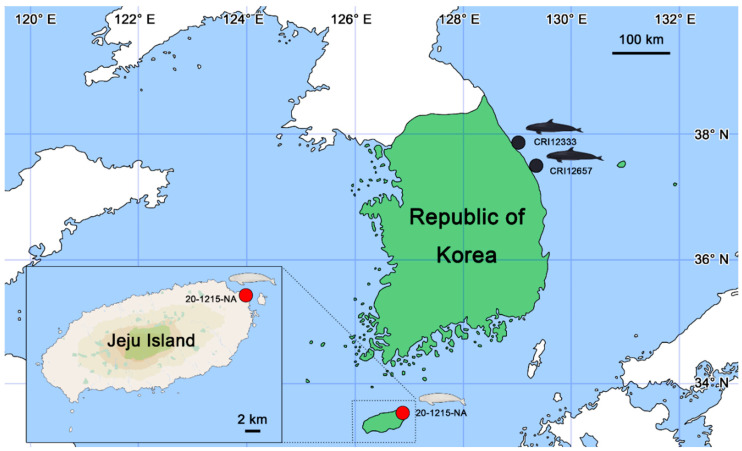
Location map of where the cetacean specimens, i.e., 20-1215-NA, CRI12333, and CRI12657 were found in the Republic of Korea (figures were illustrated by S.B.L.). A narrow-ridged finless porpoise 20-1215-NA was stranded at Hado-ri, Gujwa-eup, Jeju-si, Jeju Island, Republic of Korea (33°31′46.2″ N and 126°53′44.9″ E; red circle) on 15 December 2020. Two false killer whales, CRI12333 and CRI12657, were bycaught in nets near Gangneung (37°54′01.9″ N and 128°53′20.8″ E) on 17 November 2022 and Samcheok (37°19′16.8″ N and 129°27′02.1″ E) on 25 October 2023, respectively (black circles).

On 15 December 2020, a female narrow-ridged finless porpoise, 20-1215-NA, with a snout-to-tail length of 148.5 cm was stranded on the beach at Hado-ri, Gujwa-eup, Jeju Island, Republic of Korea (Figure 1; 33°31′46.2″ N and 126°53′44.9″ E). Age was estimated based on body length and growth layers of the teeth [32]. The carcass was stored in a −50 °C freezer for further examination. Necropsy was conducted on 1 February 2021 at the Jeju office of the Korean Fisheries Resources Agency (FIRA; 23 Ongpo 7-gil, Hallim-eup, Jeju, Republic of Korea). Anthropometric measurements and assessments of the skin and musculoskeletal system revealed multiple proliferative lesions on the skin. During necropsy, proliferative epithelial lesions of appropriate size were collected from multiple parts, including the oral and genital regions. Various organs and specimens, including the skin, muscles, stomach, lungs, kidneys, liver, lymph nodes, external genitalia, food content, and parasites, were appropriately sized and collected for molecular, histological, and pathological examinations. Samples were preserved in 70% ethanol for molecular analysis and in 10% neutral buffered formalin for histopathological analysis.

On 17 November 2022, a male false killer whale, CRI12333, with a snout-to-tail length of 326.9 cm and a weight of 359 kg, was bycaught in a fishing net in the sea near Jumunjin-eup, Gangneung, Republic of Korea (Figure 1; 37°54′01.9″ N and 128°53′20.8″ E). The carcass was stored in a −50 °C freezer for further examination. Necropsy was conducted on 15 February 2023, at the Cetacean Research Institute (CRI; 250, Jangsaengpogorae-ro, Nam-gu, Ulsan, Republic of Korea). Anthropometric measurements and skin and musculoskeletal system assessments were performed. During necropsy, genital lesions on the penis were identified and collected for examination. The organs of the respiratory, circulatory, digestive, urinary, and immune systems were isolated, and contents such as stomach food and parasites were collected and examined in detail.

On 25 October 2023, a male false killer whale, CRI12657, with a snout-to-tail length of 320.0 cm and a weight of 449 kg was bycaught in a fishing net in the sea near Samcheok, Republic of Korea (Figure 1; 37°19′16.8″ N and 129°27′02.1″ E). The carcass was stored in a −50 °C freezer for further examination. Necropsy was conducted on 13 December 2023 at the CRI. Anthropometric measurements and skin and musculoskeletal system assessments were performed. During necropsy, genital lesions on the penis were identified and collected for examination. Necropsy was performed using the same protocol as described for CRI12333.

### 2.2. Histopathology

The formalin-fixed skin samples were cut into 1.5 cm^3^ sections, including pathological and normal lesions. The samples were commissioned to Korea Vet Lab (Seongnam, Republic of Korea) to perform histological processes and analyzed at Antech Diagnostics (Fountain Valley, CA, USA). After embedding in paraffin, the tissues were sectioned into 5 µm and stained with hematoxylin and eosin (H&E).

Methenamine silver staining was performed to detect yeast [33]. The sample was washed with distilled water and then oxidized with chromic acid to produce aldehyde groups. Sodium bisulfite was added to remove residual chromic acid. After incubating with a 60 °C methenamine silver solution, gold chloride and sodium thiosulfate were added, followed by washing and conducting light-green staining.

Periodic acid–Schiff (PAS) staining was performed for hyphae [34]. The sample was oxidized for 10 min using a 1% periodic acid solution, stained for 15 min with Schiff’s reagent, and passed through a sulfurous acid solution. After washing, the nuclei were stained with hematoxylin, then decolorized with 100% alcohol, and xylene was added for clarification.

Acid-fast staining was performed to detect the bacteria [35]. Carbol fuchsin was applied to the samples, and the slides were heated using a flame. Acid alcohol was added to decolorize, a counterstain of methylene blue was applied, and then the excess stain was rinsed off.

Gram staining was performed to detect the bacteria [36]. The samples had crystal violet and iodine solution applied, and ethanol was added to decolorize them. A counterstain of safranin solution was then added, and the samples were gently rinsed.

### 2.3. Transmission Electron Microscopy (TEM)

The specimen preparation for transmission electron microscopy (TEM) was conducted as per previous protocols [37,38]. Initially, the fixed samples in 10% neutral buffered formalin underwent washing with 0.05 M sodium cacodylate buffer. Subsequently, washed samples were immersed in a 1% osmium tetroxide solution diluted in 0.1 M sodium cacodylate buffer for 1.5 h, then the samples underwent a thorough washing procedure using distilled water. The washed samples were placed in 0.5% uranyl acetate solution overnight. After washing with distilled water, the dehydration process was performed gradually using ethanol concentrations of 30%, 50%, 70%, 80%, 90%, and finally 100%. The dehydrated samples were transited into propylene oxide, 50%, 66%, and 100% Spurr’s resin solution in that order. The samples were embedded in 100% Spurr’s resin in a 70 °C incubator. Ultrathin sections (70 nm) were observed using a transmission electron microscope (80 kV) model JEM1010 (JEOL, Tokyo, Japan).

### 2.4. DNA Preparation and Sequencing

The samples in 70% ethanol were crushed physically with an Omni Bead Ruptor (Omni International, Kennesaw, GA, USA) and dissolved chemically with phosphate-buffered saline (PBS) solution and proteinase K. Total DNA products were extracted from the lesion samples using a DNeasy blood and tissue kit (Qiagen, Valencia, CA, USA). The extracted DNA products were refined with a DNeasy PowerClean Pro cleanup kit (Qiagen).

Two nested polymerase chain reaction (PCR) protocols were used to detect herpesviruses. The primers, FP1 (5′-GAY TTY GCI AGY YTI TAY CC-3′), FP2 (5′-TCC TGG ACA AGC AGC ARI YSG CIM TIA A-3′), and RP1 (5′-GTC TTG CTC ACC AGI TCI ACI CCY TT-3′), were used for the first PCR to detect DNA polymerase gene fragments 215–235 base pairs (bp) in length for most herpesviruses and 315 bp for cytomegaloviruses [17,39,40]. The PCR mixture (20 µL) included 1 µL of sample, 1 µL of each primer FP1, 5 µL of Maxime PCR PreMix (LiliF Diagnostics, Seongnam, Republic of Korea), and 11 µL nuclease-free water. PCR was carried out with the following parameters: initial denaturation at 94 °C for 2 min, 55 cycles of a denaturation step at 94 °C for 20 s, an annealing step at 46 °C for 30 s, an elongation step at 72 °C for 30 s, and a final elongation step at 72 °C for 10 min.

For the nested PCR, the FP3 (5′-TGT AAC TCG GTG TAY GGI TTY ACI GGI GT-3′) and RP2 (5′-CAC AGA GTC CGT RTC ICC RTA IAT-3′) primer pair was used. The PCR mixture (20 µL) included 2 µL of the first PCR product, 1 µL each of primers FP3 and RP2, 5 µL of Maxime PCR PreMix (LiliF Diagnostics), and 11 µL nuclease-free water. The PCR protocol for the second PCR was the same as that for the first.

Each amplified PCR product was resolved using 1.0% gel electrophoresis with 0.5 µg/mL ethidium bromide to separate the target DNA molecules. DNA fragment bands were visualized using UV transillumination. DNA fragments were extracted using a QIAquick Gel Extraction Kit (Qiagen) and sequenced for further genetic analysis at Cosmo Genetech (Seongdong-gu, Seoul, Republic of Korea). Screen gel analysis was performed with a QIAxcel Advanced Instrument (Qiagen) and QIAxcel ScreenGel Software version 1.6.

Bionics (Seongdong-gu, Seoul, Republic of Korea) conducted DNA cloning of the PCR products with a TOPcloner TA kit (Enzynomics, Daejeon, Republic of Korea), subcloned into a pTOP V2 vector, then transformed into DH5α chemically competent *Escherichia coli*. The sequences were annotated using standard nucleotide BLAST of the National Institute of Health (NIH) with standard databases and optimization for highly similar sequences (megablast) [41].

### 2.5. Phylogenetic Analysis

Phylogenetic analysis of the cetacean herpesvirus DNA polymerase gene partial fragments was performed in VICTOR [42] with the recommended settings for viruses, using 6 alphaherpesviruses (GenBank accession numbers AY608707.1, AY757301.2, AY949832.1, DQ295063.1, DQ295064.1, and KP995686.1) and 13 gammaherpesviruses (GenBank accession numbers AY949828.1, AY949830.1, AY949831.1, AY952776.1, AY952777.1, AY952778.1, AY952779.1, KP995687.1, DQ288666.1, DQ288667.1, KT591613.1, and KT991635.1) infecting cetaceans using the maximum-likelihood method after alignment with MUSCLE implemented in MEGA X with 1000 bootstraps [43].

## 3. Results and Discussion

### 3.1. Skin Lesions of Narrow-Ridged Finless Porpoise: 20-1215-NA

**Figure 2:**
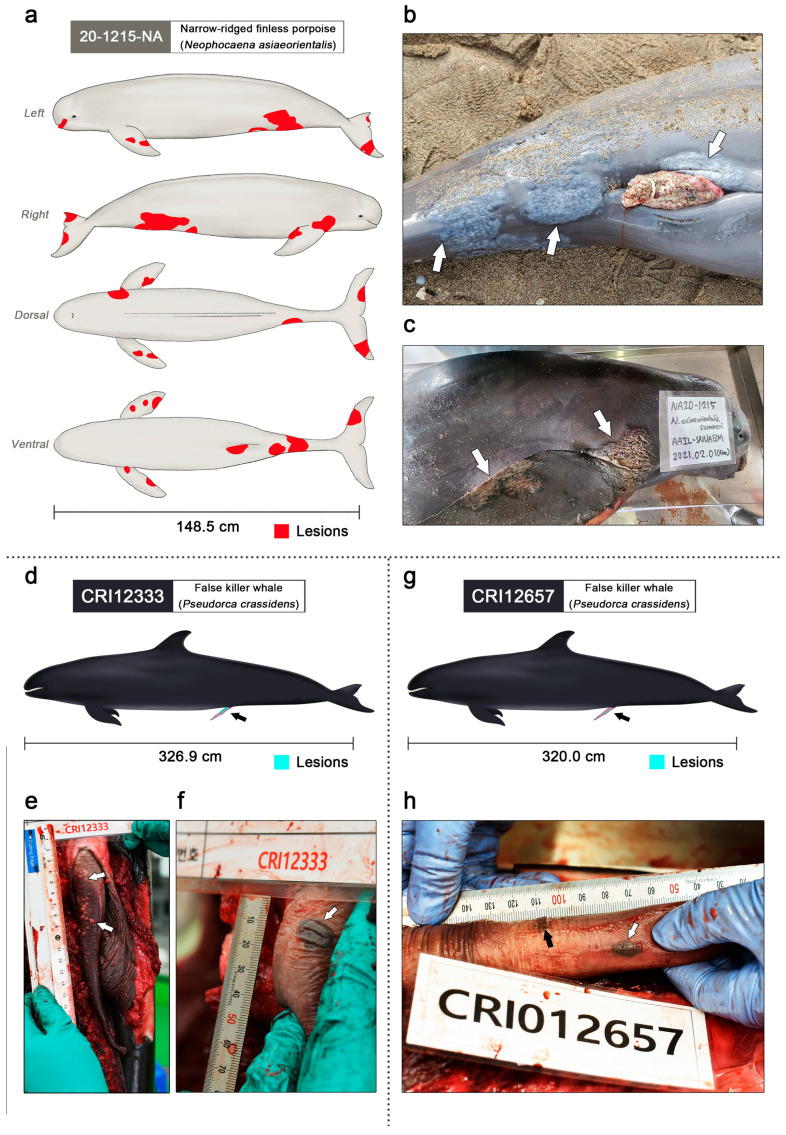
Clinical lesions of gammaherpesvirus infection in the narrow-ridged finless porpoise and two false killer whales (figures were illustrated by S.B.L.). (**a**) The left, right, dorsal, and ventral view of the narrow-ridged finless porpoise; 20-1215-NA. Observed skin lesions are marked in red. A total of twelve skin lesions were observed on the entire body. The total body length was 148.5 cm. (**b**) The largest skin lesion of 20-1215-NA was 12.8 × 11.0 cm around the genital slit region (white arrows). (**c**) Dermatitis lesions of 20-1215-NA were found on both flippers and fluke (white arrows). (**d**) Genital lesions of the false killer whale CRI12333 are marked in turquoise and indicated using a black arrow. The total body length was 326.9 cm. (**e**) Genital lesions of the false killer whale; CRI12333 (white arrows). Numerous small vesicles were scattered on the dorsal and proximal penile epidermis and within the epidermis. The diameter of the vesicular lesions varied from approximately 1 to 4 mm. (**f**) The largest genital lesion of the false killer whale CRI12333 (white arrow). The pigmented vesicular lesions were located on the ventral penile epidermis and within the epidermis, and the dimensions of the lesions were 1.5 × 1.2 cm. (**g**) Genital lesions of the false killer whale CRI12657 are marked in turquoise and indicated using a black arrow. The total body length was 320.0 cm. (**h**) Small pigmented vesicular lesions with a diameter of 1–3 mm were clustered on the ventral and proximal penile epidermis and within the epidermis (black arrows). A pigmented vesicular lesion with dimensions of 1.2 × 0.7 cm was located on the left penile epidermis and within the epidermis (white arrow).

According to dental radiography and the total body length (148.5 cm) of the finless porpoise, it was estimated that the animal was approximately 8 years old and had reached puberty. Twelve dermatitis lesions spread from the snout to the fluke at various sites on the body (Figure 2a), especially in the genital, oral, and abdominal regions (Figure 2b) including both flippers (Figure 2c). The size of the dermatitis lesions varied from 2.0 × 3.5 cm to 12.0 × 9.0 cm. The largest mucocutaneous junction (12.8 × 11.0 cm) was observed around the genital slit. The skin lesions spread in a pattern that extended from the margins of the lesions, and the affected areas were mottled and paler than the normal surrounding skin. Some lesions were cracked, sessile, and swollen, and diffused margins with cutaneous epithelial hyperplasia were present. The appearance of the lesions closely resembled the alphaherpesvirus skin infection of a captive Atlantic bottlenose dolphin (*Tursiops truncatus*), gammaherpesvirus skin infection of a harbor porpoise (*Phocoena phocoena*), and an uncertain infection of free-ranging dusky dolphins [44,45,46]. The skin lesions varied from oval to round, with protruding edges similar to those in previous cases. The epithelial layer of severe lesions peeled off, exposing the blubber. The cartilaginous tissue under the epithelium was damaged in the left ventral flipper. Ocean currents may have affected the body while floating after death. No signs of bleeding were observed in any of the lesions. The largest, and presumably the oldest, skin lesion was found around the genital slit area, whereas prominent lesions were not observed within the genital organs, including the vagina, except for the presence of vaginal prolapse. However, the possibility that systemic skin lesions originated from genital lesions cannot be ruled out. The widespread distribution of lesions throughout the body, varying lesion sizes, and large affected areas suggest that the body likely suffered from dermatitis for a considerable time. Necropsy revealed no notable symptoms except skin lesions.

### 3.2. Genital Lesions of Two False Killer Whales: CRI12333 and CRI12657

**Figure 3:**
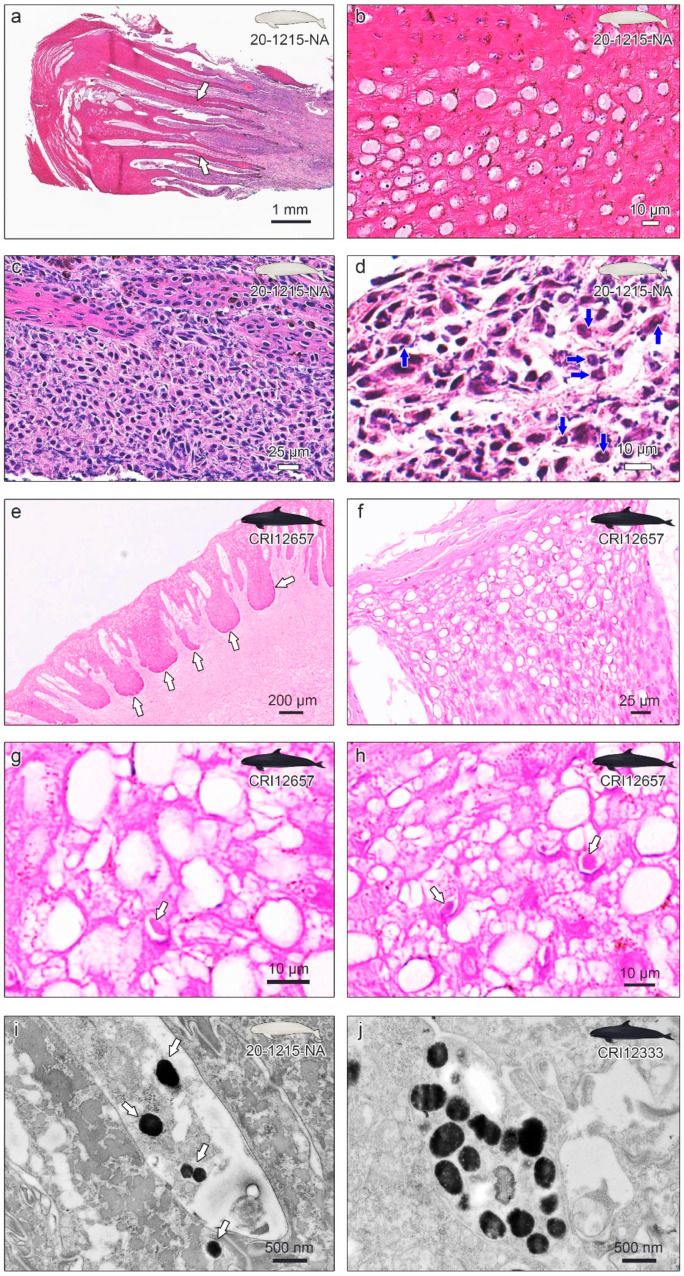
Histopathological examinations of the skin lesions of narrow-ridged finless porpoise 20-1215-NA (**a**–**d**), genital lesions of false killer whale CRI12657 (**e**–**h**), and transmission electron microscopy of the 20-1215-NA and CRI12333 lesions (**i**,**j**). (**a**) The epidermis was moderately to markedly thickened. The white arrows indicate accentuated rete pegs into the underlying connective tissue. (**b**) A ballooning change is observed with amorphous eosinophilic material in the vacuoles and moderate nuclear debris within the epidermis. (**c**) Mononuclear cells infiltrate predominantly within the dermis at the base of the proliferative epidermis. (**d**) Intranuclear eosinophilic inclusion body (INI) is observed in the epidermis (blue arrows). (**e**) The epidermis shows the forming of thick dermal papillae rete pegs. (**f**) Ballooning degeneration of many nuclei is obvious. (**g**,**h**) The eosinophilic INI was observed in the epidermis (white arrows). (**i**) Virus-like particles from the skin lesion of 20-1215-NA were observed under the transmission electron microscope (TEM). (**j**) Virus-like particles from the genital lesion of CRI12333 were observed under the TEM.

Genital lesions of the false killer whales were observed on the penis of CRI12333 (Figure 3d). Small vesicles were scattered on the dorsal and proximal penile epidermis and within the mucosa (Figure 3e). Multiple plaques above the mucosa were pale yellow with smooth surfaces and round shapes. The diameter of the vesicular plaques varied from approximately 1 to 4 mm. On the largest genital lesion of specimen CRI12333, a pigmented vesicular lesion was located on the ventral penile epidermis and within the mucosa, and the dimensions of the lesions were 1.5 × 1.2 cm (Figure 3f). The surface was tough, firm, dark, and oviform, resembling herpes viral genital lesions of bottlenose dolphins [47] and a penile lesion in a beluga (*Delphinapterus leucas*) [48]. The lesions were slightly raised with mucosal thickening, similar to those in previous cases.

Genital lesions were also observed in the penis of false killer whale CRI12657 (Figure 3g). Small pigmented vesicular lesions with a diameter of 1–3 mm were clustered on the ventral and proximal penile epidermis and within the mucosa (Figure 3h). Unlike the vesicles of CRI12333, multiple dark plaques with dried surfaces and atypical shapes were observed above the mucosa. A pigmented vesicular lesion with dimensions of 1.2 × 0.7 cm was located on the left penile epidermis and within the mucosa. The lesion resembled a pigmented plaque of CRI12333. When observing the patterns of genital infection in CRI12333 and CRI12657, it appears that small pale and yellow vesicles formed first, forming a single large plaque, along with pigmentation, as the edges of these vesicles expanded. The false killer whales CRI12333 and CRI12657 were both caught in gillnets, and necropsy findings such as foamy fluid in the respiratory system indicated that suffocation was the main cause of death [49,50].

### 3.3. Histopathological Examinations

Upon histological examinations, each skin biopsy of 20-1215-NA showed a moderately to markedly thickened epidermis with accentuated thin rete pegs (Figure 3a). In general, accentuated rete pegs are recognized in cases of acanthosis in psoriasis [51,52,53] or atopic dermatitis (AD) [54,55]. A thickened epidermis and elongated projections of epithelial cells in the submucosa were previously reported in cases of herpesvirus infection in harbor porpoises (*Phocoena phocoena*) and beluga whales (*Delphinapterus leucas*) [45,48]. There was a vacuolar change with pale, somewhat amorphous eosinophilic material in the vacuoles and moderate nuclear debris within the epidermis (Figure 3b). Some skin-infecting viruses, including herpesviruses, can cause intracytoplasmic vacuolization [56,57,58] and hypereosinophilic material [59,60]. The development of microvesicles has previously been reported in alphaherpesvirus infection in beluga whales [48,61]. Suspicious eosinophilic intranuclear inclusion (INI) bodies were identified in the epidermal cells (Figure 3c), as in previous cetacean infections [20,62,63,64]. There were moderate-to-marked infiltrates of predominantly mononuclear cells within the dermis at the base of the proliferative epidermis (Figure 3d), which is one of the histological changes observed in AD [65,66], carcinoma [67,68], and other skin diseases [69]. The non-suppurative infiltrates and INI indicated a potential viral infection [45]. Macrophages, lymphocytes, melanophages, and a few neutrophils were observed. Methenamine silver and PAS staining were negative for yeasts and fungi. Acid-fast staining did not reveal the presence of acid-fast bacteria. Compared to previous cetacean skin infection cases, the differential diagnoses for the lesions included lobomycosis caused by *Lacazia loboi*, papillomavirus, poxvirus, and possibly herpesvirus infection [17,39,40,70,71,72]. Based on the results of the histological analysis, rule-out diagnoses were performed using PCR for each possible pathogen.

Histological analysis of CRI12657 and CRI12333 genital lesions showed that the epidermis formed thick dermal papillae and rete pegs (Figure 3e). Zooming in on the epidermis, ballooning degeneration of many nuclei was evident (Figure 3f). Eosinophilic INI was observed in the epidermis (Figure 3g,h). No cellular inflammation was observed in the genital lesion samples of the CRI12333 or CRI12657 groups. Methenamine silver and PAS staining were negative for yeasts and fungi. Acid-fast staining did not reveal the presence of acid-fast bacteria. Compared to previous cetacean skin infection cases, the differential diagnoses for the lesions included lobomycosis caused by *Lacazia loboi*, papillomavirus, and possibly herpesvirus infection. Based on the results of the histological analysis, rule-out diagnoses were performed using PCR for each possible pathogen.

Comparing the histopathological results of the 20-1215-NA skin lesions and genital lesions of CRI12333 and CRI12657, the epidermis showed a common pattern of moderate-to-marked thickening with accentuated rete pegs, although there were slight differences in the degree of rete peg thickening. Vacuolar changes within the epidermis were also a distinct feature, but the 20-1215-NA sample had the additional characteristic of eosinophilic material filling the vacuoles. Furthermore, the presence and extent of the inflammatory response were a major difference between the skin and genital lesions of the two cetacean species. Porpoise 20-1215-NA with dermatitis likely experienced severe pruritus, fever, swelling, and skin sloughing due to the intense inflammatory reaction, and excessive inflammation could have even led to immunosuppression [73,74]. Given that this case involved death without clear symptoms besides widespread skin infection throughout the body, indirect anaphylactic shock by another allergen or immunosuppression could also be considered a potential cause of mortality [75]. The non-suppurative infiltrates and eosinophilic INI were also common features observed in the skin and genital lesions of the two cetacean species, strongly suggesting a herpesvirus infection.

### 3.4. Transmission Electron Microscopy (TEM)

Ultrathin sections of embedded resin block tissues were observed using transmission electron microscopy. All lesion samples of 20-1215-NA, CRI12165, and CRI12333 had herpesvirus-like particles in the epithelium layer (Figure 3i,j). These virus-like particles were irregular in shape and varied in size from 100 to 400 nm.

### 3.5. PCR, Sequencing, and Phylogenetic Analysis

**Figure 4:**
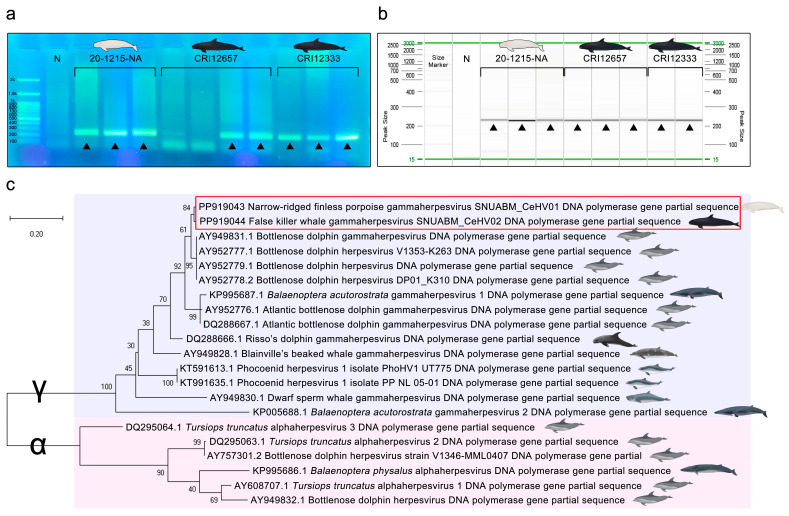
Gel electrophoresis and phylogenetic tree indicating the genetic relationship of the cetacean herpesvirus DNA polymerase gene partial sequences. (**a**) Gel electrophoresis of each PCR product. “N” indicates the negative control. Positive bands of 220 bp were observed and are indicated by black triangles (▲). (**b**) Screen gel of each PCR product. “N” indicates the negative control. Positive bands of 220 bp were observed and are indicated by black triangles (▲). (**c**) The partial sequences of the previous cetacean herpesviruses and novel cetacean gammaherpesviruses SNUABM_CeHV01 (GenBank accession number PP919043) and SNUABM_CeHV02 (PP919044) were compared. The tree was constructed using the maximum-likelihood method after alignment with MUSCLE implemented in MEGA X with 1000 bootstraps. Cetacean gammaherpesviruses (indicated by γ) are indicated by the light-blue box, and cetacean alphaherpesviruses (indicated by α) are indicated by the light-pink box.

Herpesvirus sequences were detected in all lesion samples (i.e., 20-1215-NA, CRI12333, and CRI12657) using nested PCR. The PCR products of the three specimen samples demonstrated positive bands of 220 base pairs (bp) on the gel electrophoresis (Figure 4a) and screen gel (Figure 4b), and two fragments: a 222 bp sequence of the DNA polymerase gene, showing 96.4% identity with that of a bottlenose dolphin herpesvirus (GenBank accession number AY952779.1) [17] from the skin lesion samples of the narrow-ridged finless porpoise (20-1215-NA), and a 222 bp sequence of the DNA polymerase gene, showing 95.95% identity with that of the same bottlenose dolphin herpesvirus from the genital lesion samples of the two false killer whales (CRI12333 and CRI12657). After annotation, the DNA polymerase gene partial genome sequences of the narrow-ridged finless porpoise and false killer whale gammaherpesvirus isolates, named SNUABM_CeHV01 and CeHV02, were deposited in GenBank under accession numbers PP919043 and PP919044, respectively.

Based on the DNA polymerase gene partial sequence, the two novel sequences were closely related to bottlenose dolphin gammaherpesviruses (AY949831.1, AY952777.1, AY952778.1, and AY952779.1) from bottlenose dolphins [17], and secondly related to *Balaenoptera acutorostrata* gammaherpesvirus 1 (KP995687.1) from common minke whales (*Balaenoptera acutorostrata*) [76] and Atlantic bottlenose dolphin gammaherpesviruses (AY952776.1 and DQ288667.1) [17] from Atlantic bottlenose dolphins (Figure 4c).

Herpesviruses have host-specific susceptibility owing to their host cell receptor specificity, dependence on intracellular replication processes and specificity of immune evasion strategies [77,78,79]. Given these characteristics, it was expected that the phylogenetic relationships among cetacean species and the relationships among the herpesvirus strains infecting these species would show some degree of correlation. However, the phylogenetic tree of the DNA polymerase genes revealed a completely different pattern. For example, the narrow-ridged finless porpoise is a species of the Phocoenidae family, which includes the harbor porpoise (*Phocoena phocoena*) and the other six Phocoenidae species [80,81]. However, Phocoenid herpesvirus 1 (KT591613.1 and KT991635.1) found from harbor porpoises [45] was not closely related to the narrow-ridged finless porpoise gammaherpesvirus SNUABM_CeHV01 identified in this study (Figure 4). The correlation between cetacean herpesviruses and cetacean species was insignificant.

Gammaherpesvirus infections have been reported in skin lesions of various vertebrates. For example, gammaherpesviruses have been identified in the proliferative skin lesions of the South American fur seal (*Arctocephalus australis*), fisher (*Martes pennanti*), and sheep (*Ovis aries*) [82,83,84]. Meanwhile, a few gammaherpesviruses have been identified in cetaceans, and these viruses mostly infect genital lesions, lymph nodes, or the central nervous system [17,58,85]. Skin infections caused by gammaherpesviruses have rarely been reported in cetaceans. Only Phocoenid HV1 has been identified in the cutaneous lesions of harbor porpoises (*Phocoena phocoena*) [45]. In this study, we focused on a novel gammaherpesvirus, SNUABM_CeHV01, which infects the skin of narrow-ridged finless porpoises. Although the skin of porpoises belonging to the Phocoenidae family may be more susceptible to gammaherpesvirus infection than the skin of other cetacean species, further studies should be conducted to understand gammaherpesvirus pathophysiology in cetaceans.

A representative characteristic of the family Herpesviridae is latent infection, which makes molecular diagnosis difficult [86,87,88]. In Herpesviridae, the target organs for latency vary depending on the subfamily. Gammaherpesviruses are highly lymphotropic, alphaherpesviruses infect neurons latently, and betaherpesviruses have variable tropism [89]. Owing to latent infections, herpesviruses have also been detected in several cetacean cases without clear symptoms or manifestations [85].

This study highlights the need to detect the latent presence of herpesviruses in healthy narrow-ridged finless porpoises and other cetaceans. Numerous narrow-ridged finless porpoises and false killer whales may be latently infected with herpesvirus in South Korean seawater. Cetacean herpesviruses are mostly associated with sexually transmitted diseases that can negatively affect the sexual behavior and distribution of animals.

The narrow-ridged finless porpoise populations have declined dramatically over the last decade [29,30]. This study confirmed the presence of cetacean herpesviruses in South Korean waters. As these viruses can potentially impact the breeding and conservation of endangered species, it is imperative to further monitor and screen for herpesviruses in other cetacean species in South Korean waters. The pathophysiology of cetacean herpesviruses should be investigated further to conserve the porpoises and other cetacean species.

## 4. Conclusions

This study focused on gammaherpesvirus infection in three individuals: a narrow-ridged finless porpoise (20-1215-NA) with severe and clear skin dermatitis and two false killer whales (CRI12333 and CRI12657) with penile lesions. This study amplified and detected partial sequences of the DNA polymerase genes of SNUABM_CeHV01 and CeHV02 and observed the associated lesions to confirm the clinical signs of viral infection. The main histopathological findings included accentuated rete pegs, ballooning changes, and eosinophilic intranuclear inclusion (INI) bodies. The limitations were that the viral DNA fragments were extracted from biopsies of deceased individuals, resulting in relatively short base-pair lengths of the sequence fragments, and the researchers were unable to conduct a full genome analysis of the viruses.

However, the significance of this study lies in the fact that it demonstrated the existence of novel cetacean herpesviruses and through phylogenetic analysis gained an understanding of the relationship between these newly identified viruses and other known cetacean herpesviruses. This represents an important step forward in studying potentially harmful pathogens affecting endangered whale and dolphin populations.

## Data Availability

The DNA polymerase gene partial genome sequences of the narrow-ridged finless porpoise and false killer whale gammaherpesvirus SNUABM_CeHV01 and CeHV02 were deposited at GenBank under the accession numbers PP919043 and PP919044, respectively.
